# Association of *NRG1* and *AUTS2* genetic polymorphisms with Hirschsprung disease in a South Chinese population

**DOI:** 10.1111/jcmm.13498

**Published:** 2018-01-29

**Authors:** Yan Zhang, Xiaoli Xie, Jixiao Zeng, Qiang Wu, Ruizhong Zhang, Deli Zhu, Huimin Xia

**Affiliations:** ^1^ Department of Pediatric Surgery Guangzhou Institute of Pediatrics Guangzhou Women and Children's Medical Center Guangzhou Medical University Guangzhou Guangdong China

**Keywords:** Hirschsprung disease, association, epistasis, subclinical stratification

## Abstract

Hirschsprung disease (HSCR) is a genetic disorder characterized by the absence of enteric ganglia. There are more than 15 genes identified as contributed to HSCR by family‐based or population‐based approaches. However, these findings were not fulfilled to explain the heritability of most sporadic cases. In this study, using 1470 HSCR and 1473 control subjects in South Chinese population, we replicated two variants in *NRG1* (rs16879552, *P* = 1.05E‐04 and rs7835688, *P* = 1.19E‐07), and further clarified the two replicated SNPs were more essential for patients with short‐segment aganglionosis (SHSCR) (*P* = 2.37E‐05). We also tried to replicate the most prominent signal (rs7785360) in *AUTS2*, which was a potential susceptibility gene with HSCR. In our results, in terms of individual association, marginal effect was observed to affect the HSCR patients following recessive model (*P* = 0.089). Noteworthy, significant intergenic synergistic effect between rs16879552 (*NRG1*) and rs7785360 (*AUTS2*) was identified through cross‐validation by logistic regression (*P* = 2.45E‐03, OR = 1.53) and multifactor dimensionality reduction (MDR, *P* < 0.0001, OR = 1.77). Significant correlation was observed between expression of these two genes in the normal segments of the colons (*P* = 0.018), together with differential expression of these genes between aganglionic colonic segments and normal colonic segments of the HSCR patients (*P* value for *AUTS2* <0.0001, *P* value for *NRG1 *=* *0.0243). Although functional evaluation is required, we supply new evidence for the *NRG1* to HSCR and raised up a new susceptibility gene *AUTS2* to a specific symptom for the disease.

## Introduction

Hirschsprung's disease (HSCR) is a developmental disease with strong genetic components characterized by the absence of enteric ganglia. The incidence of HSCR varies in different races; it is roughly three times frequent among Asians (approximately 1 in 5000 newborns) comparing with Europeans (1 in 15,000 newborns) 1. Although the familial aggregation exists (up to 20% of the cases), the disease mainly presents sporadically 2. The patients can be classified according to the aganglionosis length into short (SHSCR), long (LHSCR) and total colonic aganglionosis (TCA) with the percentages around 80%, 15% and 5%, respectively. In very rare cases, patients were affected with the total intestine aganglionosis (TIA) 3.

To date, rare variants in more than 15 genes were identified as contributed to HSCR, centred by the most frequently mutated *RET* 4. However, these rare variants were not fulfilled to explain such big proportion of sporadic cases. Genome‐wide association studies (GWAS) have identified several genes including *NRG1* at 8p12 and *SEMA3* at 7q21 in East Asians and Europeans, respectively 5. Subsequently, follow‐up replication study of these loci reveals the consistent genetic associations in different cohorts 6, 7, 8, 9. Similar with the findings in *RET*, the congregation of both common and rare variants in *NRG1* underlies the predisposition to HSCR 10, 11. Recently, Jiang *et al*. 12 performed a meta‐analysis on NRG1, showing SNPs rs7835688 and rs16879552 were more likely to serve as associated variants specifically in Asians, and they further clarified these two SNPs may affect different subclinical symptoms, which required further validation in independent cohort. Limited by the sample size and incomplete clinical records in previous studies, the functional mechanisms by the identified HSCR‐associated variants were unclarified; there are also several potential‐associated susceptibility loci that were listed and awaited for further validation in independent cohorts.

In this study, using 1470 HSCR cases and 1473 controls, we conducted a replication study of two SNPs in HSCR‐associated gene, *NRG1* to evaluate its stratified function to disease and one SNP in HSCR candidate gene, *AUTS2* to confirm the association with disease. The association of *NRG1* was further replicated in our study. Consistent with the findings in *RET*, the common variants of *NRG1* mainly explained the association in SHSCR patients. We failed to further support the individual association of *AUTS2* with disease. Surprisingly, in congregation with the associated SNPs at *NRG1*, the variant on *AUTS2* elevated the risk to HSCR. This finding may partially explain the missing heritability so far to the disease.

## Material and methods

### Study subjects

The samples in this study were recruited from Guangzhou Women and Children's Medical Center. The study was approved by the institutional review board of the hospital. Written informed consents were provided by guardians of all subjects. All the cases were diagnosed with HSCR by surgical procedures and followed up histological examination. A total of 1470 patients were recruited from 2000 and were claimed as South Chinese divided by the aganglionic status including short segment (SHSCR, 1033 cases), long segment (LHSCR, 294 cases) and TCA (82 cases), respectively. The subclinical information was closely examined (Table S1). The blood samples of 1473 controls matched ethnically and geographically were collected with no history of HSCR and neurological developmental disorders.

### Selected SNP Genotyping and quality control

The three SNPs involved in study were selected according to the first published HSCR GWAS study 13; the most prominent signals in the established loci and potential loci were listed in the Supplementary files of the study. Two SNPs in NRG1 were selected following the two listed criteria: (*i*) SNP surpassing the genome‐wide association significance (*P* < 1 × 10^−8^). 2. The two SNP showed limited LD (*r* ^2 ^< 0.5). The prominent signals in potential loci were selected according to the minor allele frequency no less than 5% in Chinese population (CHB) in public database. Quality control of the three SNPs was performed as follows: (*i*) All the three SNPs surpassed the filtering standard with missing call rate no bigger than 10%. (*ii*) Any subjects with 10% missing call were removed. After quality control, all three SNPs were kept for further analysis consisted of 1469 cases and 1466 controls.

### Association analysis and subphenotype stratification

The SNPs were analysed for association with the disease by comparing the risk of allele frequency (allelic test) in patients and controls as well as other tests using PLINK 1.9 (additive test by logistic regression, Cochran–Armitage trend test, test of dominant and recessive models, genotype test of 3 × 2 contingency tables)^14^. Association stratified by subphenotype was analysed by comparing cases with a certain subphenotype with controls.

### Independence testing

Linkage disequilibrium (LD) patterns and values were obtained using Haploview 15. SNPTEST v2.5b was used to perform the logistic regression tests in this study 16. Tests of independent contributions towards disease associations for SNPs in a single locus were carried out using logistic regression, adjusting for the effect of a specific SNP in the same locus.

### Genetic epistasis

Epistasis test (case–control analysis) by logistic regression was adopted here for parametric analysis of genetic interaction using PLINK1.9 14. PLINK uses a model according to allele dosage ranging from 0 to 2, indicating the number of risk alleles for each SNP, A and B, and fits the model in the form of Y = b0 + b1 SNPA + b2 SNPB + b3 SNPA × SNPB + e. The parameters b1, b2 and b3 indicate the contribution of SNP A and SNP B and interaction between A and B. The test for interaction is based on the coefficient b3. *P* value of <0.05 was considered statistically significant.

Pairwise nonparametric epistasis test was also applied using MDR analysis 17. This method includes a combined cross‐validation (CV)/permutation testing procedure that minimizes false‐positive results by multiple examinations of the data. The statistical significance was determined by comparing the average prediction error from the observed data with the distribution of average prediction errors under the null hypothesis. The MDR analysis was carried out using version 2.0 of the open‐source MDR software package that is freely available online (http://www.epistasis.org).

### Real‐time PCR

Colon total RNA from HSCR patients was isolated using the ‘iScript TM cDNA Synthesis Kit' (170‐8891; Bio‐Rad, Hercules, California, USA), and under standard conditions, equal amounts of each sample were reverse‐transcribed into cDNA by the action of the iScript reverse transcriptase. The real‐time PCR was carried out using a *QuantStudio*™ 6 Flex sequence detector (Applied Biosystems, Centre Drive Foster City, California, USA). In each reaction, 25 ng cDNA was amplified in a 20‐μl volume using the iTaq Universal SYBR^®^ Green Supermix (172‐5124; Bio‐Rad). The PCR conditions were 95° for 1 min. followed by 40 cycles of 95° for 15 sec. and 60° for 1 min. GraphPad 5.0 (GraphPad Software, Inc., La Jolla, California, USA) was used to test the tissue‐specific expression difference using pairwise *t*‐test and expression correlation using Spearman correlation test.

## Results

### Association of NRG1 and AUTS2 SNPs with HSCR

We selected two identified SNPs on *NRG1* and the most prominent SNP on *AUTS2* for replication in 1470 cases and 1473 controls from South Chinese population. The genotype distribution for the three SNPs followed the Hardy–Weinberg equilibrium (HWE) in the subjects (*P*_hwe = 0.76 for rs7785360, *P*_hwe = 0.26 for rs16879552, *P*_hwe = 0.26 for rs7535688). As shown in Table 1, all three SNPs are located in the intronic region. For the SNP rs7785360 in *AUTS2* gene, we failed to replicate the association in our population (*P *=* *0.149, OR = 1.14), and marginal effect was observed in the recessive model of association with disease (*P *=* *0.089, OR = 1.19). The minor allele was concordant with the previous GWAS study 13; thus, the trend was not present, and we are not sure whether the trend is consistent between two studies. The association of SNP rs16879552 and rs7835688 was replicated with milder effect size (1.23 *versus* 1.68, 1.43 *versus* 1.98), comparing this study and the first GWAS study 13. To better digest the effective pattern for the two SNPs on *NRG1*, we specified the samples following four different genetic models including additive, dominant, recessive and genotypic models. Larger effect was observed in both SNPs following recessive models (OR = 1.47 for rs16879552 and OR = 2.62 for rs7835688, respectively). The association of the three SNPs was further examined, adjusting the potential effect by sex, and as shown in Table S2, we observed consistent results with Table 1. HaploReg databases 18 integrating ENCODE and other data were used to enable the regulatory and epigenomic annotation onto the three SNPs selected for replication and SNPs with high LD (*r* ^2 ^> 0.8) (Fig. S1). According to HaploReg, the replicated SNP rs7835688 was found as an expression quantitative locus (eQTL) which may affect the expression of *NRG1*. SNPs showed high LD (*r* ^2 ^> 0.8) with rs16879552 and rs7785360 was also suggested as eQTL to *NRG1* and *AUTS2,* respectively. All the SNP locates within DNase I hypersensitive regions reported in multiple different cell types, and histone modification markers such as H3K27ac and H3K9ac. Moreover, the variants also alter several transcription factors binding motifs. The information may highlight the potential functional roles of the replicated SNPs, which is waiting for further functional characterization.

**Table 1:** Replication results of three SNPs on *NRG1* and *AUTS2* in South Chinese population using 1470 cases and 1473 controls

CHR	SNP	BP	A1/A2	Gene	Feature	Left_gene	Right_gene	TEST	Case	Control	OR (CI 0.95)	*P*
*P_hwe *= 0.7559												
7	rs7785360	69944392	G/A	*AUTS2*	intron [NM_015570.1]	STAG3L4	WBSCR17	Freq	91.88%	90.82%	1.14 (0.95–1.37)	0.149
								ADD	2659/235	2650/230	1.14 (0.95–1.37)	0.152
								DOM	1433/14	1448/11	0.77 (0.35–1.72)	0.534
								REC	1226/221	1202/257	1.19 (0.97–1.44)	0.089
								GENO	1226/207/14	1202/246/11	NA	0.143
*P_hwe *= 0.2604												
8	rs16879552	32553698	C/T	*NRG1*	intron [NM_013964.2]	LOC100127894	MST131	Freq	48.60%	43.45%	1.23 (1.11–1.37)	1.05E‐04
								ADD	1349/1427	1247/1623	1.23 (1.11–1.37)	1.13E‐04
								DOM	1008/380	987/448	1.20 (1.02–1.42)	0.025
								REC	341/1047	260/1175	1.47 (1.23–1.77)	3.02E‐05
								GENO	341/667/380	260/727/448	NA	1.12E‐04
*P_hwe *= 0.2575												
8	rs7835688	32553981	C/G	*NRG1*	intron [NM_013964.2]	LOC100127894	MST131	Freq	22.10%	16.60%	1.43 (1.25–1.63)	1.19E‐07
								ADD	633/2231	483/2427	1.42 (1.25–1.62)	1.72E‐07
								DOM	551/881	450/1005	1.40 (1.20–1.63)	2.09E‐05
								REC	82/1350	33/1422	2.62 (1.74–3.95)	4.43E‐06
								GENO	82/469/881	33/417/1005	NA	2.52E‐07

CHR, chromosome; SNP, single‐nucleotide polymorphism; BP, base pair of where the SNP is located. Func.refgene, the function role of SNP in the gene; Gene.refgene, the gene where the SNP located to; A1/A2 indicates the risk allele and protective allele to disease; Freq indicates risk allele frequency of the SNP in cases or controls. ADD, DOM, REC and GENO indicate the association test following additive, dominant, recessive and genotypic models. The *P* value indicates the significance based on different genetic models. The calculation of odds ratio (OR) is also based on the risk allele of each SNP.

### Independence testing of NRG1 SNPs

To further identify the relationship between the replicated two SNPs on *NRG1*, the LD patterns were examined on our replication data and public available data including East Asians and Caucasians (Fig. S2). The LD in East Asian populations and our study was similar and showed moderate LD between two SNPs (*r* ^2 ^= 0.37 in East Asian, *r* ^2 ^= 0.27 in current study). Limited LD was detected between the two SNPs in Caucasians (*r* ^2 ^= 0.01). These results give us hint that the two associated variants may derive from two different casual variants. Pairwise independence test of the two SNPs was performed using logistic regression by controlling the effect of one of the two SNPs (Table 2). SNP rs7835688 kept significant after controlling the effect of rs16879552 (*P* = 1.35E‐04, OR = 1.36). However, SNP rs16879552 remains no significance to disease if the effect of rs7835688 was controlled (*P* = 0.208, OR = 1.08). It seems that the independence of SNP rs7835688 to disease was without any concern. However, for SNP rs1679552, the independence to disease was not identified in our study, which may due to the sample size limitation. It is also possible that the SNP might exist diversified effect to disease such as genetic interaction.

**Table 2:** Independence test by adjusting for the effects of other SNPs in the *NRG1* region

SNP	SNP whose effect was adjusted^a^	
	rs16879552	rs7835688
rs16879552	NA	*P *=* *0.208
		OR = 1.08 (0.96–1.23)
rs7835688	***P *** **=** *** *** **1.35E‐04**	NA
	**OR = 1.36 (1.16**–**1.59)**	

a The data in each column represent the remaining effect of association (*P*‐values) after adjusting for the effect of SNP(s) on the top row of each column. SNPs with *P* value surpassing statistical significance (0.05) were boldfaced.

### Intergenic SNPs show epistatic effect to HSCR

The SNPs can influence the disease risk individually (main effects) or behave jointly. We use pairwise epistatic analysis implemented by PLINK to test the three SNPs genotyped in this study. As shown in Table 3 (right top), the result suggested significant elevated epistatic effect between SNP rs16879552 (*NRG1*) and rs7785360 (*AUTS2*) to disease (*P* = 2.45E‐03, OR = 1.53), and the detailed summary of each individual SNP fitting the logistic regression models was listed in Table S3, showing the epigenetic effect is larger than the association of each individual SNP. The SNP rs7835688, which showed strong individual association and independent effect, also showed marginal inter/intragenic epistatic effect with rs7785360 (*AUTS2*,* P *=* *0.076) and rs7835688 (*NRG1*,* P *=* *0.063), respectively. The epistatic significance supported by logistic regression was further validated using MDR analysis. Table 3 (left bottom) showed the results of balanced accuracy (BA) and the results of cross‐validation consistency (CVC) of the two‐locus model. The significance of the result was tested, showing the consistent higher effect size between epistatic pairs SNP rs16879552 (*NRG1*) and rs7785360 (*AUTS2*) to disease (*P* < 0.0001, OR = 1.77). The detailed risk genotype combinations were shown in Figure S3 generated by MDR. Consistent with the risk genotypes of rs16879552 (CC) and rs7785360 (GG) for individual SNP associations, the combination CC‐GG performed a significant higher risk to disease by chi‐square test (*P* = 3.01E‐08).

**Table 3:** Pairwise epistatic interacting results among three variants in *NRG1* and *AUTS2* carried out by logistic regression and multifactor dimensionality reduction (MDR)

		*AUTS2*	*NRG1*	
SNP	Interaction	rs7785360	rs16879552	rs7835688
		Logistic regression		
rs7785360	MDR	NA	***P*** ** = 2.45E‐03**	*P* = 0.076
			**OR = 1.53 (1.16**–**2.02)**	OR = 1.36 (0.97–1.91)
rs16879552		CVC = 10, BA = 0.543	NA	*P* = 0.063
		OR = 1.77 (1.46–2.14), *P* < 0.0001		OR = 1.25 (0.99–1.59)
rs7835688				NA
	

OR means odds ratio for interaction, and a value of 1.0 indicates no effect. Cross‐validation consistency (CVC) reflects the number of times MDR analysis identified the same model as the data were divided into different segments. Balanced accuracy is defined as (sensitivity +specificity)/2. SNPs with *P* value surpassing statistical significance (0.05) were boldfaced.

### Clinical stratification of SNPs in NRG1 and AUTS2 with HSCR

HSCR is a heterogenous disease, and different patients may be diagnosed with varied clinical manifestation, such as the aganglionosis lengths of the colon. The association of HSCR patients specified by different affected length *versus* controls was listed as shown in Table 4. For the SNP rs7785360 in *AUTS2*, it is more likely to affect the LHSCR patients although the effect is still marginal (*P *=* *0.06, OR = 1.40). Further replication is still required to confirm the association with disease. For the two replicated SNPs in *NRG1*, the association of SNPs with disease was aggregated in the SHSCR patients, with a larger effect size (OR = 1.28 for SNP rs16879552, OR = 1.47 for SNP rs7835688) through subphenotype‐control analysis. Consistent with the common variants identified in *RET,* the associated variants are more likely to affect SHSCR patients. Surprisingly, inconsistent with the findings on SNP rs16879552, SNP rs7835688 also showed strong association with TCA patients (*P *=* *9.77E‐03, OR = 1.62). We further examined the association of SNP with subclinical stratification including enteritis before and after operation, gender, by case‐only testing. No significant findings were identified based on current study (data not shown). Upon the epistatic effect of SNP rs7835688 (*NRG1*) and rs7785360 (*AUTS2*) to HSCR, we further specified whether the trend was kept in different subclinical groups. As shown in Table S4, we observed significant epistatic associations in SHSCR and LHSCR patients with the disease, but not in TCA patients which may be caused by limited samples waiting for further replications.

**Table 4:** Association results of three SNPs in *NRG1* and *AUTS2* to different subclinical features classified by aganglionosis length including short length (SHSCR), long length (LHSCR) and TCA

CHR	SNP	A1/A2	Gene	Length of aganglionosis	TEST	Case	Control	OR (CI 0.95)	*P*
7	rs7785360	G/A	AUTS2	SHSCR (1033 cases and 1473 controls)	Freq	91.36%	90.82%	1.07 (0.88–1.31)	0.51
					ADD	964/978	2650/268	1.40 (0.98–1.98)	0.51
					DOM	716/255	1448/11	2.18 (0.28–16.95)	0.2
					REC	248/723	1202/257	1.41 (0.97–2.03)	0.29
					GENO	248/468/255	1202/246/11	NA	0.17
				LHSCR (294 cases and 1473 controls)	Freq	93.23%	90.82%	1.39 (0.98–1.97)	0.06
					ADD	537/39	2650/268	1.40 (0.98–1.98)	**0.06**
					DOM	287/1	1448/11	2.18 (0.28–16.95)	0.46
					REC	250/38	1202/257	1.41 (0.97–2.03)	0.07
					GENO	250/37/1	1202/246/11	NA	0.18
				TCA (82 cases)	Freq	92.35%	90.82%	1.22 (0.68–2.18)	0.5
					ADD	157/13	2650/268	1.23 (0.68–2.19)	0.5
					DOM	85/0	1448/11	NA	NA
					REC	72/13	1202/257	1.18 (0.65–2.17)	0.58
					GENO	72/13/0	1202/246/11	NA	NA
8	rs16879552	C/T	NRG1	SHSCR (1033 cases and 1473 controls)	Freq	49.64%	43.45%	1.28 (1.14–1.44)	**2.37E‐05**
					ADD	1862/176	1247/1623	1.10 (0.91–1.32)	**2.49E‐05**
					DOM	1006/13	987/448	1.08 (0.81–1.43)	**8.77E‐03**
					REC	856/163	260/1175	1.21 (0.88–1.66)	**1.31E‐05**
					GENO	856/150/13	260/727/448	NA	**3.49E‐05**
				LHSCR (294 cases and 1473 controls)	Freq	45.71%	43.45%	1.10 (0.91–1.32)	0.32
					ADD	256/304	1247/1623	1.10 (0.91–1.32)	0.32
					DOM	197/83	987/448	1.08 (0.81–1.43)	0.6
					REC	59/221	260/1175	1.21 (0.88–1.66)	0.25
					GENO	59/138/83	260/727/448	NA	0.5
				TCA (82 cases)	Freq	45.12%	43.45%	1.07 (0.78–1.47)	0.67
					ADD	74/90	1247/1623	1.07 (0.78–1.48)	0.67
					DOM	58/24	987/448	1.10 (0.67–1.79)	0.71
					REC	16/66	260/1175	1.10 (0.62–1.92)	0.75
					GENO	16/42/24	260/727/448	NA	0.91
8	rs7835688	C/G	NRG1	SHSCR (1033 cases and 1473 controls)	Freq	22.61%	16.60%	1.47 (1.27–1.69)	**1.25E‐07**
					ADD	458/1568	483/2427	1.46 (1.27–1.68)	**2.03E‐07**
					DOM	393/620	450/1005	1.42 (1.20–1.68)	**5.18E‐05**
					REC	65/948	33/1422	2.96 (1.93–4.53)	**6.57E‐07**
					GENO	65/328/620	33/417/1005	NA	**1.12E‐07**
				LHSCR (294 cases and 1473 controls)	Freq	20.04%	16.60%	1.26 (1.00–1.58)	0.05
					ADD	113/451	483/2427	1.27 (1.01–1.60)	0.05
					DOM	100/182	450/1005	1.23 (0.94–1.60)	0.13
					REC	13/269	33/1422	2.08 (1.08–4.01)	0.03
					GENO	13/87/182	33/417/1005	NA	0.06
				TCA (82 cases)	Freq	24.39%	16.60%	1.62 (1.12–2.35)	**9.77E‐03**
					ADD	40/124	483/2427	1.67 (1.14–2.45)	**8.77E‐03**
					DOM	39/43	450/1005	2.03 (1.30–3.17)	**1.99E‐03**
					REC	1/81	33/1422	0.53 (0.07–3.94)	0.54
					GENO	1/38/43	33/417/1005	NA	**3.61E‐03**

SNPs with *P* value surpassing statistical significance (0.05) were boldfaced.

### The mRNA expression of NRG1 and AUTS2 in HSCR colon samples

**Figure 1:**
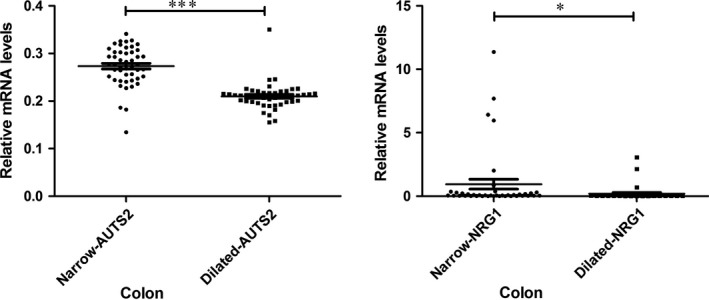
Tissue‐specific differential expression of *NRG1* and *AUTS2* in aganglionic and dilated (normal) colons of the HSCR patients.

To further evaluate the presence and distribution of the identified two susceptibility loci including *NRG1* and *AUTS2*, we collected tissue samples from 54 HSCR patients to pairwise compare the expression level of targeted gene in the aganglionic segments (narrow) and normal segments (dilated) using qRT‐PCR as shown in Figure 1. We observed consistent higher expressions of *NRG1* (*P* = 0.0243) and *AUTS2* (*P* < 0.0001) in aganglionic segments of patients comparing with the normal segments through paired *t*‐test. To gain further biological insight, we examined the expression of *NRG1* and *AUTS2* in both aganglionic segments and normal segments. We found that *NRG1* expression was highly correlated with the expression of *AUTS2* in the normal segments, suggesting a potential functional link between these two genes (Spearman *r* = 0.36, *P* = 0.0176). There is a much weaker expression correlation in the aganglionic tissues between these two genes (Pearson's *r* = −0.16, *P* = 0.34). In summary, we detected much stronger expression correlation between *NRG1* and *AUTS2* in normal tissues than in disease tissues.

## Discussion

HSCR is a polygenic disease; most studies were designed on association studies through case–control study or trio study. In attempt to find additional loci contributing to the disease, it is applicable to use larger sample size with detailed clinical records to survey suggestive association loci in previous study. Benefit from GWAS findings, in this study through a total number of 1470 cases and 1473 controls matched geographically and ethnically, we selected two SNPs on susceptibility gene *NRG1* for the subclinical stratification analysis, pointing to the elevated risk to SHSCR comparing to other disease status. We also chose a SNP for further replication on *AUTS2*, and the individual association of the selected SNP was only concentrated on patients with long‐segmental aganglionosis. Surprisingly, we observed a boost predisposition to disease if the intergenic epistasis between SNPs on *AUTS2* and *NRG1* was considered.

NRG1 is a membrane glycoprotein which plays a critical role in the growth and development of multiple organ systems 19. The dysregulation of this gene has been reported to other neurological diseases such as schizophrenia 20, 21, 22. In addition, rare variants were subsequently identified as contributed to HSCR. However, for most of the sporadic cases, rare variants were seldom supplied to explain the disease manifestation. Common variants are still the most appropriate markers to further bridge the links between clinical symptoms and genetic predisposition. In current study, for the first time, we identified the GWAS‐identified SNP rs16879552 and rs7835688 are more likely to affect the patients with short‐length aganglionosis following a recessive pattern. Although these two SNPs showed moderate LD, no study was working on the relationship between two established SNPs. As we presented in Table 2, the independence contribution of rs16879552 was not identified. It might be caused by limited sample size in this study and large impact difference between the two SNPs (three magnitude differences in terms of association *P* value). In principle, it is also possible that the association of two SNPs was derived from one causal variant. However, we identified inconsistent patterns in terms of the subclinical feature correlation between two SNPs. For SNP rs7835688, in addition to the elevated risk to SHSCR patients which is similar to SNP rs16879552, it is also contributed to the higher risk to the TCA patients (*P* = 9.77E‐03, OR = 1.62). It can be explained by the small sample size of the TCA patients, which may lead to false positive. It is also possible that the two common SNPs serve in different manner to the disease. It should be noticed that the test for subclinical symptoms is before correction for multiple testing and the sample size among SHSCR, LHSCR and TCA patients was unbalanced, which may lead to false‐positive discovery and false‐negative results. An independent replication work in the same cohort, especially in the life‐threatening LHSCR and TCA ones, would substantially help to clarify the disease mechanisms and further to improve clinical intervention.

*AUTS2* has been implicated in neurodevelopment; it is reported to be involved in numerous central nervous systemic disorders, including intellectual disability and developmental delay 23. However, limited studies were concentrated on the enteric nerve system until the HSCR GWAS study 13. In this study, we found marginal individual association of rs7785360 with disease, especially in LHSCR patients. Interestingly, most of the study so far confirmed the impact of common variants to disease was contributed to SHSCR, including our findings in *NRG1*. The findings on *AUTS2* may partially fill the missing pieces of puzzle to explain the aetiology of HSCR. In this study, we observed two SNPs in *NRG1* (rs7835688) and *AUTS2* (rs7785360) may play unknown roles to severe cases of HSCR patients.

Integrative investigation by Gui *et al*. 24 proved the interaction of variants in *RET* and *NRG1* increases the risk to HSCR development. In our previous study, we also proved the intragenic epistasis in *RET* common variants elevated the risk to HSCR 25. Epistasis between the same or different genes provides us a new perspective for exploring hidden genetic influence. As mentioned by Gui *et al*. 24, it is relatively more difficult to explore the interaction effect of two binary covariates when the sample size is not big, which usually needs 1000 or above samples to reach the 80% power. Taking the advantage of large replication samples, we tested the pairwise genetic epistasis between *NRG1* and *AUTS2*. A significant synergetic interaction between rs16879552 (*NRG1*) and rs7785360 (*AUTS2*) was identified through the CV by logistic regression and MDR analysis. We examined the colonic mRNA expression of *NRG1* and *AUTS2* in both aganglionic segments and dilated segments, showing consistent higher expressions of *NRG1* and *AUTS2* in the aganglionic segments compared with dilated segments. The expression correlation of *NRG1* and *AUTS2* was also detected, and we found the high expression correlation in dilated colonic segment was broken down in the aganglionic colonic segment. Rieger and colleagues also found *NRG2* and *AUTS2* showed evidence of coexpression through microarray data 26. *NRG1* and *NRG2* belong to the same family sharing one EGF‐like domain, necessary and sufficient for binding to and activating ERBB receptors 27. We further speculated the expression correlation across different tissues between *NRG1* and *AUTS2* through public available database. Similar to our results in normal colonic tissue, *NRG1* was found to have high expression correlation with *AUTS2* (*r* ^2 ^= 0.4) (Fig. S4)^28^. This piece of data gave us hint that NRG1 and AUTS2 may cooperate which might affect the normal status of human under the trigger of unclear mechanism, like genetic interaction. However, the characterization of regulatory SNPs still suffers from incomplete understandings on functionally important motives. The functional impact of natural variants on a given trait is one of the most pressing questions in genetics. Further *in vitro* study should be designed to determine the functional impact of replicated SNPs and validated each of these genetic interactions in terms of functional influence on the disease.

In summary, although the functional mechanisms subject to the association of *NRG1* and *AUTS2* for disease is unclear, we identified the two common variants in *NRG1* were associated with HSCR risk in South Chinese population, especially in the SHSCR patients. We also found common variants in *NRG1* and *AUTS2* were elevated the risk of severe cases of HSCR patients, which was complementary with the common variants in *RET* gene. The boosted risk to HSCR through genetic interaction of associated variants between *NRG1* and *AUTS2* shed novel light on fully understanding of the aetiology of this genetic complex disease.

## Conflict of interest

The authors declare that they have no conflict of interest.

## Supporting information


**Fig. S1** The functional annotation of three replicated SNPs and SNPs with *r* ^2 ^> 0.8 in AUTS2 and NRG1 reflecting potential epigenetic and expression regulation roles.
**Fig. S2** The LD structure of two SNPs in *NRG1* in different populations.
**Fig. S3** The optimal two‐locus model as determined by multifactor dimensionality reduction analysis on variants in NRG1 and AUTS2.
**Fig. S4** The Coexpression correlation between NRG1(Gene ID 3084) and AUTS2 (Gene ID 26053) normalized across different organs from COXPRESdb ver. 6.0.
**Table S1** The clinical stratification of the subjects in this study.
**Table S2** Replication results of three SNPs on *NRG1* and *AUTS2* in South Chinese population using 1470 cases and 1473 controls conditioning on the gender difference.
**Table S3** The summary statistics of individual SNPs in the interaction model for SNP pair rs7785360 and rs16879552.
**Table S4** Pair‐wise epistatic interacting results among three variants in *NRG1* and *AUTS2* done by logistic regression and Multifactor dimensionality reduction (MDR)subclassified by the aganglionosis length of the patients.
