# Supplementary material for: Association of NRG1 and AUTS2 genetic polymorphisms with Hirschsprung disease in a South Chinese population
Source: J Cell Mol Med. 2018 Jan 29;22(4):2190–9. doi: 10.1111/jcmm.13498 (PMC5867083; doi:10.1111/jcmm.13498)
Supplement: Supplementary file 1 — Fig. S1 The functional annotation of three replicated SNPs and SNPs with r 2 > 0.8 in AUTS2 and NRG1 reflecting potential epigenetic and expression regulation roles. Fig. S2 The LD structure of two SNPs in NRG1 in different populations. Fig. S3 The optimal two‐locus model as determined by multifactor dimensionality reduction analysis on variants in NRG1 and AUTS2. Fig. S4 The Coexpression correlation between NRG1(Gene ID 3084) and AUTS2 (Gene ID 26053) normalized across different organs from COXPRESdb ver. 6.0. Table S1 The clinical stratification of the subjects in this study. Table S2 Replication results of three SNPs on NRG1 and AUTS2 in South Chinese population using 1470 cases and 1473 controls conditioning on the gender difference. Table S3 The summary statistics of individual SNPs in the interaction model for SNP pair rs7785360 and rs16879552. Table S4 Pair‐wise epistatic interacting results among three variants in NRG1 and AUTS2 done by logistic regression and Multifactor dimensionality reduction (MDR)subclassified by the aganglionosis length of the patients. [file JCMM-22-2190-s001.docx]

**The file includes four Supplementary figures and four Supplementary tables.**

**Supplementary Figure 1. The functional annotation of three replicated SNPs and SNPs with r2>0.8 in AUTS2 and NRG1 reflecting potential** **epigenetic and expression regulation roles**


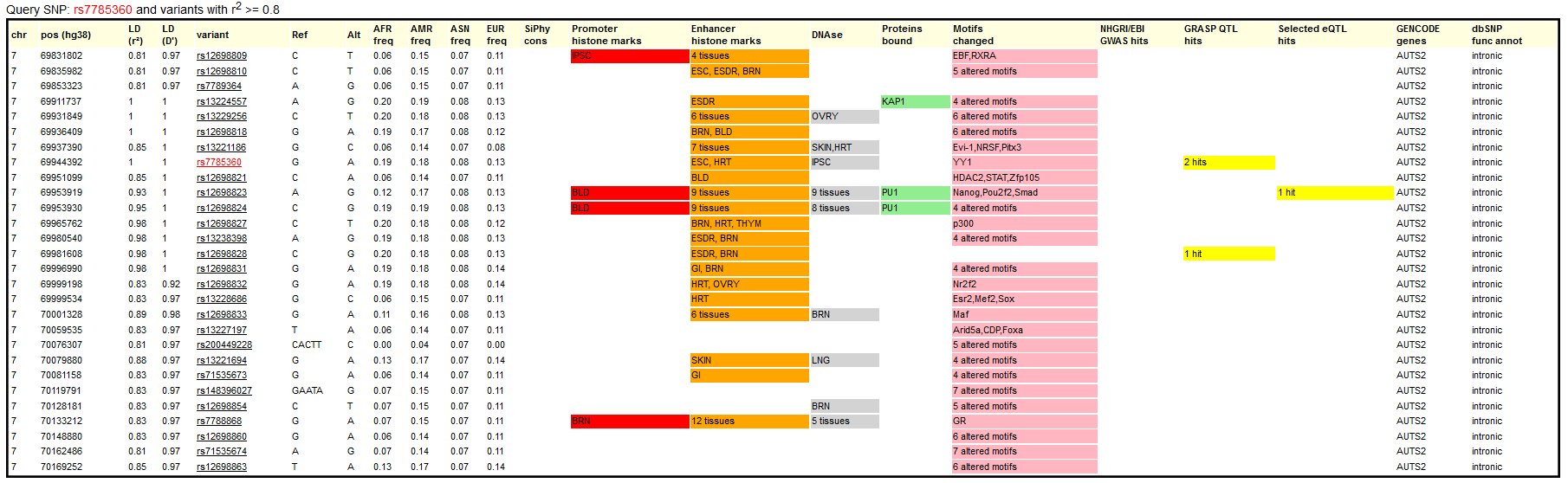


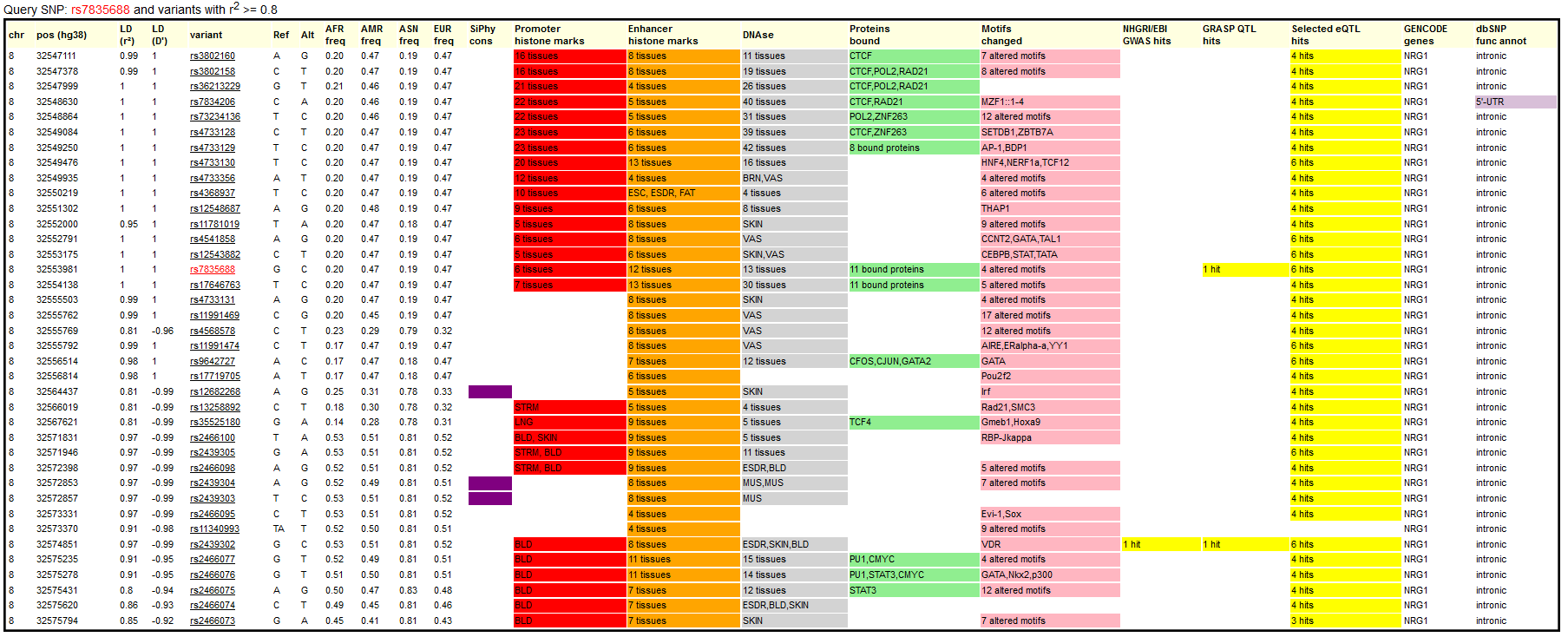


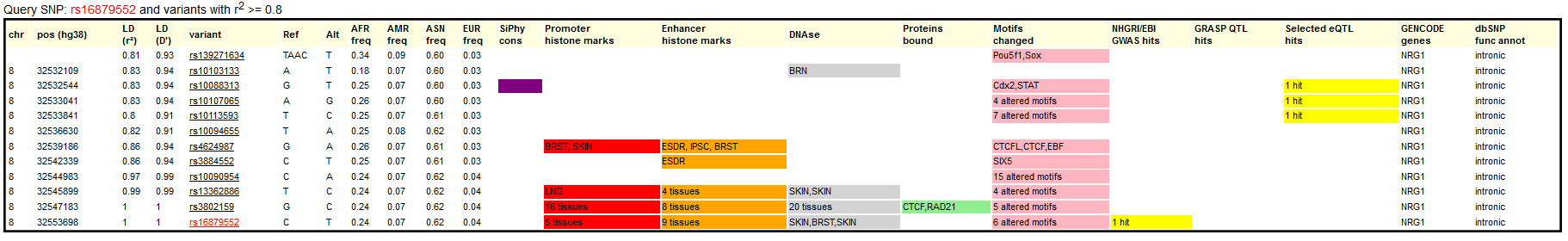


Resource: http://archive.broadinstitute.org/mammals/haploreg/haploreg.php

**Supplementary Figure 2. The LD structure of two SNPs in *NRG1* in different populations.**


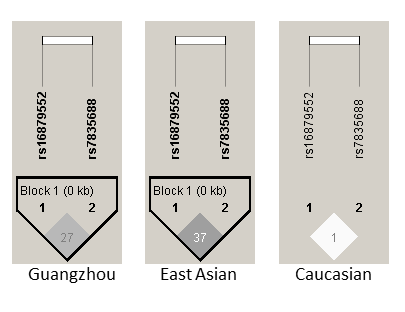


**Supplementary Figure 3.** **The optimal two-locus model as determined by multifactor dimensionality reduction analysis on variants in NRG1 and AUTS2.** The numbers within each small square represent the number of individuals with the speciﬁc genotype combinations (di-genotypes) in cases (left) and controls (right). The dark-shaded squares indicate risk for disease, whereas light shaded squares represent low risk for disease. (Samples with missed call were negelected for plotting the square)


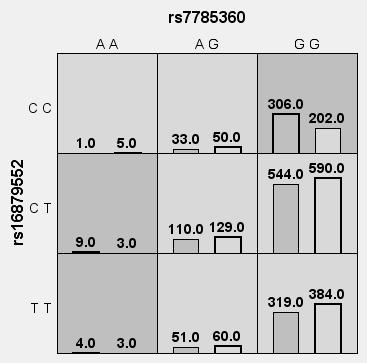


**Supplement Figure 4. The Coexpression correlation between NRG1(Gene ID 3084) and AUTS2 (Gene ID 26053) normalized across different organs from COXPRESdb ver. 6.0.**

**
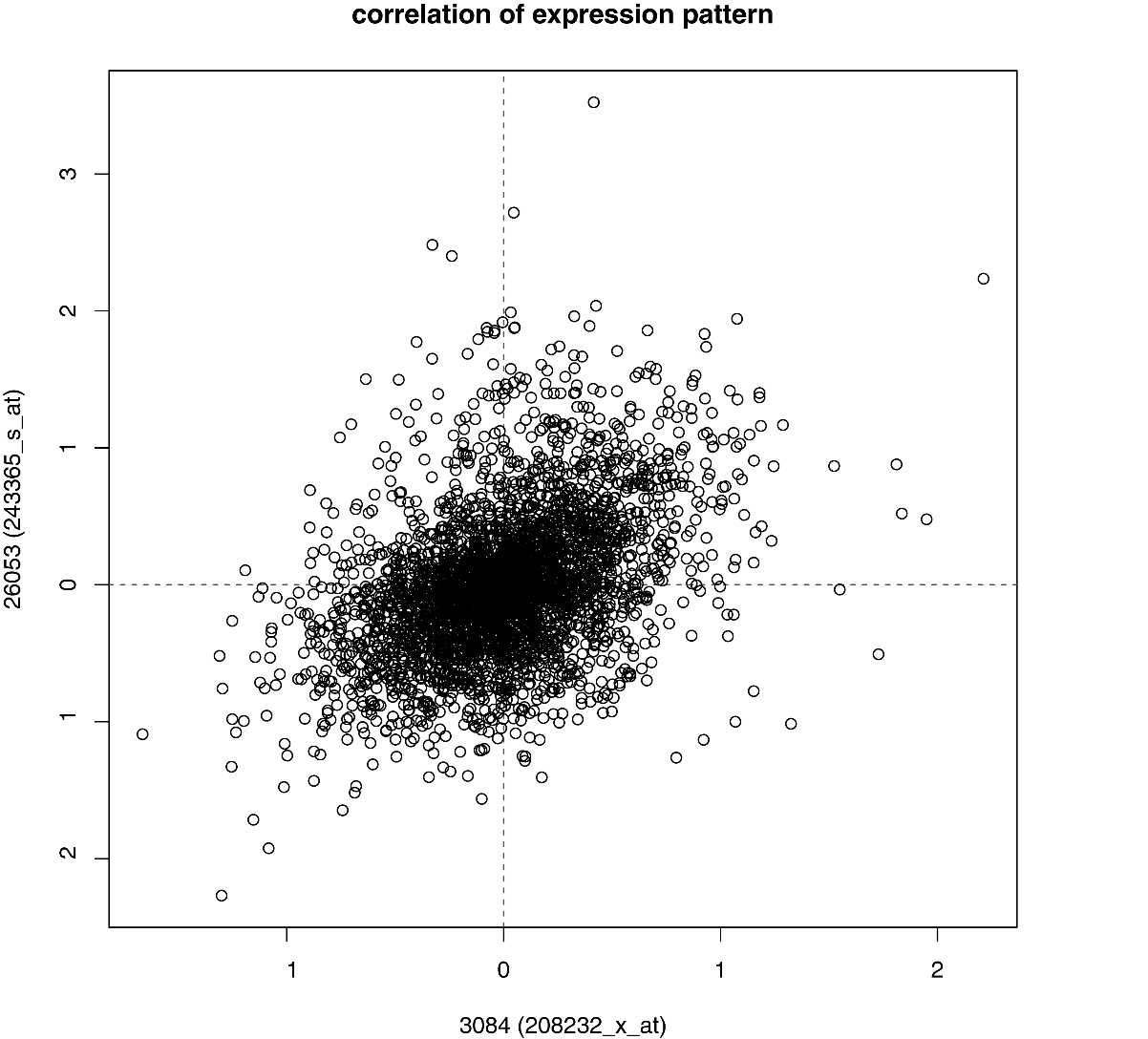
**

**Supplementary Table 1. The clinical stratification of the subjects in this study.**

|  |  |  |  |  |
| --- | --- | --- | --- | --- |
| HCSR subphenotype | Cases (n=1470) | % | Controls (n=1473) | % |
| Subjects |  | | | |
| Age range（Months） | 8.37±20.50 |  | 18.61±19.75 |  |
| ≤2 | 725 | 49.32% | 458 | 31.09% |
| >2 | 745 | 50.68% | 1015 | 68.91% |
| Gender |  | | | |
| Females | 240 | 16.33% | 967 | 65.65% |
| Males | 1230 | 83.67% | 506 | 34.35% |
| Clinical manifestation |  | | | |
| SHCSR | 1033 | 70.27% |  | |
| LHCSR | 294 | 20.00% |  |  |
| TCA | 82 | 5.58% |  |  |
| Total intestine | 3 | 0.20% |  |  |

**Supplementary Table 2. Replication results of three SNPs on *NRG1* and *AUTS2* in South Chinese population using 1470 cases and 1473 controls conditioning on the gender difference.**

| CHR | SNP | A1 | OR | P* |
| --- | --- | --- | --- | --- |
| 7 | rs7785360 | G | 1.13(0.94~1.36) | 0.21 |
| 8 | rs16879552 | C | 1.21(1.09~1.35) | 4.26E-04 |
| 8 | rs7835688 | C | 1.40(1.22~1.60) | 1.18E-06 |

P* the association of SNP to HSCR conditioning on the gender of samples.

**Supplementary Table 3. The summary statistics of individual SNPs in the interaction model for SNP pair rs7785360 and rs16879552.**

| SNP | A1 | OR | P |
| --- | --- | --- | --- |
| rs7785360 | A | 1.30(0.95~1.79) | 0.1025 |
| rs16879552 | C | 1.33(1.19~1.49) | 9.13E-07 |
| rs7785360 x rs1687955 | AXC | **1.53(1.16~2.02)** | 2.45E-03 |

**Supplementary Table 4. Pair-wise epistatic interacting results among three variants in *NRG1* and *AUTS2* done by logistic regression and Multifactor dimensionality reduction （MDR）subclassified by the aganglionosis length of the patients.**

| SNP | Interaction | *AUTS2* | | | | *NRG1* | | | | | |
| --- | --- | --- | --- | --- | --- | --- | --- | --- | --- | --- | --- |
|  |  | rs7785360 | | | | rs16879552 | | | rs7835688 | | |
|  |  | Logistic regression | | | | | | | | | |
|  |  | | SHSCR | LHSCR | TCA | SHSCR | LHSCR | TCA | SHSCR | LHSCR | TCA |
| rs7785360 | MDR | | NA | | | **P= 0.019** | **P=0.020** | 0.6281 | P= 0.120 | P= 0.207 | P=0.9171 |
|  |  |  |  |  |  | **OR=1.43 (1.06~1.92)** | **OR=1.90 (1.10~3.26)** | OR=1.24 (0.52~2.93) | OR= 1.33 (0.93~1.92) | OR=1.56 (0.78~3.09) | OR= 1.05  (0.40~2.77) |
| rs16879552 |  |  | **CVC=10, BA=0.5599** | **CVC=10, BA=0.5478** | CVC=10, BA=0.5377 | **NA** | | | P=0.070 | P=0.335 | P=0.446 |
|  |  |  | **OR= 2.00(1.65~2.42), P<0.0001** | **OR=1.74(1.30~2.34), P=0.0002** | OR=1.38(0.85~2.22), P=0.1889 |  |  |  | OR=1.27  (0.98~1.64) | OR=1.23  (0.81~1.87) | OR= 0.77 (0.39~1.51) |
| rs7835688 |  |  |  |  |  |  |  |  | NA | | |
|  |  |  |  |  |  |  |  |  |  |  |  |

OR means odds ratio for interaction, and a value of 1.0 indicates no effect. Cross-validation consistency (CVC) reflects the number of times MDR analysis identified the same model as the data were divided into different segments. Balanced accuracy is defined as (sensitivity + specificity)/2.

**Notes:**

**The raw genotype files can be supplied upon request, limited by the file size.**
